# A Lecture, Delivered in King's College, London, on Tuesday, the 11th of October, 1831: Being Introductory to the First Botanical Course of the Session Opening the Institution

**Published:** 1832-10

**Authors:** Gilbert T. Burnett

**Affiliations:** Professor of Botany in the College, and Fellow of several Societies.


### BOTANY.

*A Lecture, delivered in King's College, London, on Tuesday, the* 11*th of October,* 1831: *being introductory to the first Botanical Course of the Session opening the Institution.* By Gilbert T. Burnett, Professor of Botany in the College, and Fellow of several Societies.

Gentlemen: Meeting for the first time within these walls, for the first time treading on this, now classic, ground, it is natural to expect that the first thoughts conceived, the first words spoken, should be those of congratulation. Yes, now, if ever, may all concerned in the establishment of King's College be well permitted a moment's pause to indulge their feelings of honest pride, in contemplating the progress of a work like this; a work so noble in its design, so grand in its execution, and so incalculably beneficent in its anticipated results. Based on the fear of God, and built for the love of man, we cannot doubt that with such a beginning, thus continued, our system of instruction will conduce to temporal good, and tend to happiness eternal.

Gentlemen: on an occasion like the present, when our doors have been so lately for the first time opened, and our threshold for the first time passed by those students who now throng our halls; when the patrons and friends of this noble institution have assembled, even at this early hour, to encourage by their presence the exertions of those, to whom its future care has been committed; I feel that I should be wanting in duty to them, I should be wanting in respect to my office and myself, were I, as if on an ordinary occasion, to proceed without preface to the business of my course; and to neglect an opportunity, which can never occur again, of giving vent to those sensations that all must feel, and mingling my heartfelt congratulations with those which I hear echoing on every side around me. At an epoch like the present, I repeat, some more general observations than at any other period would be allowed, seem to be not only relevant, but required.

For common occasions, common congratulations would suffice; but how unfit does ordinary language seem to celebrate the extraordinary events of an age like this: when wisdom, which so long has been despised and rejected by the many, is urged on their attention by the ardor of the few; when all those treasures of science, which through such dangers have been guarded, and such labours have been earned, by the liberal, the enlightened, and the thoughtful, are now freely opened, and as freely offered, nay, not only given, but forced on the acceptance of the prejudiced, the ignorant, and the thoughtless; given by those who know their value, to such as too often esteem them not.

The restoration of knowledge as their birthright to the many, which so long they had forsaken that it became the heritage of the few, is indeed a noble act, and one worthy no mean congratulation. It is a boon, however, which, at an earlier epoch, might possibly have been bestowed in vain; nay more, it would probably have proved not a blessing, but an occasion of falling. May the power of knowledge, now so generally entailed, be ever hereafter rightly used; may its importance be always duly felt; and, to feel its worth, let them who have it, think of their state who have it not; and further, let them who *will have it,* but who *now have it not,* bethink them, *when they have it,* that once *they had it not.*

Gentlemen. Wisdom, our most excellent gift, is too often, and has been too long, considered, as something abstruse and difficult of attainment, as arduous in the pursuit, and repulsive when obtained; and thus philosophy, the desire or love of wisdom, has too lightly been esteemed, and too commonly denounced as a passion little suited to the welfare of the world in general, and only fit to be indulged by those who, forswearing its pleasures, its allurements, and its cares, withdraw from intimate communion with their species, and, scarcely remaining within the pale of society, become no longer of much service either to their kind or to themselves. Philosophers, in accordance with this error, have too frequently been esteemed peculiar and eccentric individuals, who, dreaming by day as well as by night, are bewildered in the mazes of thriftless speculation, and engaged in the hopeless task of straining after knowledge as far beyond human reach as it would be found unfit, if gained, to further the happiness of man.

Here, however, it would be a needless task to tell, that such are not philosophers, that such is not philosophy. I stand not here the apologist of what neither needs to be defended nor excused: I seek but to remove a prejudice, to reconcile the froward to so great a good; to shew that true wisdom is not only valuable when obtained, but pleasing in the acquirement; that it is not only desirable as an end, but delightful in its means.

The greatest philosophers are still but men by nature, and ever must remain but men in knowledge. They differ no more from their fellow-men than do such as see with just perceptions from such as behold without perceiving; no more than do such as seek the sun of science from such as, shrouding themselves in Cimmerian shades, believe all things invisible they see not.

Man is an inventor, not a creator; he finds, he does not form; and philosophy is the handmaid, not the ancestor of truth. She does not make the facts that she reveals, nor do the deeds that she discloses: her utmost is to raise the shroud, or rend the veil of ignorance, and point to that which already is, to that which always has been, to that which always will be; to those realities which always have been, are, and will be, whether we know them or know them not. And should she seem ever to prefer a bitter boon, her followers may rest assured it is like the bitterness of Tobit's gall, by which the scales of mental blindness may be shed; or by which may be absorbed the "drop serene,' more dangerous by far, because, more subtile and invisible itself, it makes more surely all invisible within.

Hence, as leading to a more just estimation of science, all true philanthropists will esteem it a matter of fervent congratulation, that "philosophy no longer is confined to the study and the cloister, but walks abroad amongst the busy haunts of men;" frequents our most public ways, and rears her splendid fanes on the banks of this our noblest river, and by the side of one of our most public streets. It is truly a subject for no slight congratulation that now it is no longer considered "unphilosophic, and the height of ill-breeding in scholarship, to vulgarise science, by rendering it intelligible and useful;" that philosophers no longer are compelled to veil knowledge in mystery, as men no longer are content to admire the most what least they understand, but science is esteemed according to its practical utility.

These, and many other features peculiar to our age, and which this institution is so well calculated to encourage and correct, are meet subjects for no common congratulation; but, grateful as the pursuit of such a theme would be, I check myself abruptly; for, after the dedicatory service, the address, and the lectures of my colleagues, to which you have already listened with so much interest and attention, and which must be still fresh in your memory, if not almost sounding in your ears, I am conscious that my feeble voice would scarcely now be heard, my humble meed be praiseless; and yet I will venture to affirm, that tribute by none would more willingly be given, were the mite but worthy of acceptance. Hence, feeling that the best offering I can make, will be a conscientious performance of my duty, I shall turn at once to the business of the course, and close these prefatory remarks by breathing a fervent hope, that the principles you have so lately heard laid down, the advice so ably and so kindly given, will be always, and by all observed; for should any of us, rejoicing in the pride of life, and trusting to our own strength alone, swerve from the strict path of duty, we shall surely fall; fall, perhaps, never to rise again: or, if to rise, arise shorn of our strength, like Sampson from the arms of Delilah.

I quit then, as it becomes me, all such general topics, and leave them in far abler hands: by our most reverend diocesan and learned principal, their tenor has already been in part explained: and, computing from the data thence derived, we boldly may foretell that this Institution, founded on the most philanthropic basis, (always referring to that only true philanthropy, the united love of God and man,) supported by the most patriotic zeal, (always distinguishing zeal from zealotry, true patriotism from false parade;) and conducted on the most liberal principles, (always meaning by these terms freedom, not licentiousness, liberality, not libertinism,) will confer a blessing on the present generation, and descend to future ages a glorious testimony of the pious care, munificence, and wisdom of its illustrious founders. Who, while engaged in the pleasing task of planting science in this soil, which is fruitful to a proverb, (for what does not London nurture, even to excess, whether of good, to pre-excellence in worth, or of evil, to the contrary sad extremity in crime,) have so fenced round their tree of knowledge, that its branches may not be warped by sophistry, nor its buds blighted to disease by scepticism, hoping thus, by careful culture, to ensure an abundant crop of the fruits of good, not evil; and desiring rather by vigilance to exclude, than by severity to extirpate those deformities which negligence would undoubtedly encourage and suffer to increase: and who thus have shewn that, whatever others do, they do not dare to pave the road to the tree of knowledge, and neglect the path which leads to the tree of life.

Would truth alone dispense, then, indeed, we might rest content with Plato, that "knowledge were but remembrance." On the contrary, however, knowledge is often to be made by oblivion; and, to possess a clear and warrantable body of truth, we must forget and part with much we know. Yet this is no easy task: how doubly hard do we find it to forget those things which we wish not to remember! Were it not better, then, as it were easier far, that they had been never learned? for, as Sir THOMAS BROWNE most truly adds, "such errors as are but acorns in our younger brows, become sturdy oaks in our elder heads, and resist inflexibly the powerfulest arm of reason."

Can such a system merit man's reproach? if so, reproach be thou my lot; for such reproach should ever be esteemed more truly praise.

But to our theme.

Gentlemen: Botany, the professed object of these lectures, is a science which relates to all those inferior ranks of the organic creation called PLANTS, or *Vegetables.*

But what is a plant? what do we mean by this word vegetable? It is a term the most ignorant presume they understand, although the most learned are unable exactly to define: for a plant is, indeed, as Theophrastus long ago observed, "a various thing, of which it is difficult to give a definition."

Tell a clown that it is difficult to distinguish between an animal and a plant, he will smile incredulously, and perhaps will say, can I mistake man-orchis roots for men? but shew him a conferva and a polype, a lichen and a coralline, a flustra and a flag, or even a mushroom and a mollusc, and he will at once confess, at least by silence, if not by words, that he "kens not which they be."

Such presuming self-confidence in what they know, is the "badge of ignorance and the curse of fools;" it is the humble privilege of the wise alone to doubt; and they who know the most are always the most sensible how little the most enlightened know.

But this matter is apocryphal not to the unlearned and the ignorant alone: physiologists the most astute have laboured, and do labour, still in vain, succinctly, yet comprehensively, to define a plant. The difficulty, however, consists not so much in the perception of the differences which undoubtedly do exist, as in reducing these perceptions of the progressive scale of creation to our still very imperfect language. The dilemma somewhat resembles that in which an ancient philosopher is said to have been involved, who, when desired to state what motion is, after much consideration, rose from his seat, walked towards the inquirer, and replied, "You see it, I can shew it to you, but I cannot tell you what motion is." Thus, also, to our opening question I would answer, here are plants; you see them; I can shew them to you, even if I cannot, at this early period of our course, precisely tell you what a vegetable is.

Let not the bearing of this statement, however, by any one be misunderstood. Remember it is not science which *makes* the difficulty she here points out; she only shews what already is: just as "a microscope does not make the hairs on a mite's back, but only brings them within our sphere of vision." Examine for a moment these specimens illustrative of the different departments of the vegetable world; these mushrooms, flags, and mosses; these jointed and these jointless ferns; these grasses, sedges, rushes, lilies, palms; these pines, and forest trees; and these more showy flowering herbs and shrubs; of each of which extensive sections, but meagre examples, can, of necessity, be brought before you, and yet which are scattered in such infinite profusion "o'er all the deep green earth," that their varied forms and beautiful appearances are familiar to the least observant: examine these, and say, do they not attest the dogma of him of old that a vegetable is, indeed, a various, a very various thing, of which it is difficult to give a definition: and do they not equally proclaim that science does not make the difficulty she here points out? do they not declare that she only shews what already is, although it may have hitherto escaped our observation? And hence we may conclude that the unlearned do not know more truly, because they are insensible of the imperfections of their knowledge, any more than a road becomes smooth to the purblind, merely because they do not see its roughness. Whatever is, still is, whether we know it or know it not; doubtless from the beginning eight planets always were, although the ancients knew but seven; for Herschel's telescope did not create the Georgium Sidus, but only shewed to man what mortal eyes had never seen before.

But the difficulty of diagnosis between animals and plants, and even between living and lifeless beings, so often and by so many dwelt on, is rather a speculative than a practical obscurity. Every one is sensible of differences existing between the numerous productions of nature; for were not such differences obvious, the whole would be esteemed not various, but the same. All persons, then, distinguish the peculiarities that mark the successive grades of physical existence, though few are competent to state precisely in what that difference consists: the one is the unsought observation of the savage, the other the hard-earned achievement of the sage; the former a perception that no one can avoid, the latter a science in which, not seldom, the wisest are at fault.

Now this great and extraordinary variety, this almost infinite diversity, in the structure and functions, the characters and appearances, the properties and purposes, of plants, which renders it so difficult to frame a concise definition rigidly including the whole, and as rigidly excluding all that *we think* not plants; which circumstance so many have bewailed, and which some superficial philosophers have regarded as the reproach of Botany, because it suits not their weak and artificial systems of arrangement, so far from being an "opprobrium botanicum," is in truth one of the chief advantages of which the science has to boast; and, if I wished for a change at all, I would wish (although it is needless) that the variety were ten times greater: for though the vegetable kingdom, by stretching to such wide extremes, may render the absolute definition of a plant somewhat abstruse and difficult; and, though in some few cases, at the confines of the animal and vegetable reigns, doubts may arise as to whether certain microscopic beings are animals or plants, belong to this kingdom or to that, or in fact to either, still their ambiguity, which has been lamented as a disadvantage, when rightly viewed, becomes at once an index to elucidate the things themselves; and their very obscurity indicates at once their station, by referring them to the debatable land of natural existence; just as green is neither blue nor yellow, but in the bow of heaven holds an intermediate place. And furthermore, it, of course, will follow that, the greater the diversity among decided plants, the stronger will the contrasts be; and, of course, the more easily will they be distinguished from each other; a secondary advantage, which far outweighs any slight inconvenience attending the diffuseness of the primary definition.

Still, before we presume to talk of plants, it may perhaps be required that we should attempt to solve the question that so continually recurs; viz. what is a vegetable? For plants are the principles on which all botanic lore depends; they are the very subject-matter upon which we must discourse: and, although we cannot absolutely, we can relatively, define them, which relative definition is, in truth, all that can legitimately be sought in any branch of natural history or philosophy. With this relative definition we shall, therefore, rest content; for the search after the absolute and positive too often becomes, as Butler has observed, on a somewhat similar occasion,

"An ignis fatuus that bewitches,
And leads men into pools and ditches."

Hence, to shew what constitutes this various thing we call a vegetable; i. e. to indicate the various phenomena exhibited by certain physical existences, to note what characters distinguish the organic from the inorganic world; and amongst organic beings the vegetable, or merely vital, from the animal or sensual, creation; in a word, which constitutes the several grades of men, brutes, and plants, as it involves much curious and important knowledge, shall find a place in this course of lectures.

But, for the present, let the speculative problem pass: to it we shall return hereafter; and its consideration is only now delayed, that a previous practical demonstration of plants, as they are found in nature, may better enable us to venture on its solution. Hence to a future lecture we postpose our definition of a plant, and propose in this, as a most useful preliminary step, to practically shew what a varied thing a vegetable is.

Here you must grant me, what will, however, scarcely be denied, (for it is a postulate without which no step can be advanced,) viz. that the examples adduced are truly vegetables: I have endeavoured to select as specimens those which will the best illustrate the varied characters of plants: that they are truly such I shall prove to you hereafter; that they are what I describe them you must grant me now.

Something must be assumed in every science, and the pupil must take much, at first, on the authority of the teacher; but, although it is convenient, in order that every point, even the simplest, should receive its due share of consideration, to assume that students are totally ignorant of the subject to be discussed, still such *tabulæ rasæ* are never met with; many things are unavoidably known to almost all, our very existence convinces us of many: and from the certainty of things already known, we proceed to inform ourselves respecting those which are as yet unknown; and not only this, but from knowledge thus acquired, we are enabled to correct those errors by which, either from ignorance or prejudice, we had been previously enthralled.

To this practical solution of the problem what is a vegetable, let me, therefore, entreat your earliest attention; for familiar as science and art, working by the head of the botanist and the hands of the gardener, have rendered the inhabitants of this island with vegetables in almost all their multiplied diversities, (for, besides the productions of its natural fertility, our country has long blushed with flowers not its own,) I am still inclined to think that even the most common and the most strongly marked types and classes to which I have referred will be recognised more surely by the exhibition of specimens, be they never so homely, than by reference to them by words alone, be the language never so precise. Hence, to aid verbal descriptions, which of necessity must ever be imperfect, I have collected, and here present you with, some few samples of the regions and classes, and of several of the subordinate departments, of the vegetable reign; most of which have been long established; and, although their names at first may have been waywardly imposed, custom has now rendered them more or less current terms, and science, if she has scrupled to adopt the popular language, has not scrupled to avail herself of popular observations, and has frequently adopted and confirmed the popular distribution, as a reference to these tables and diagrams will shew; and as, with very slight modifications, many of these common and familiar words may be rendered synonymes of less familiar technicalities, I shall use them now in preference; not, however, as superseding the terms of science, but as paving the way for their introduction.

| | | | | | |
|---|---|---|---|---|---|
| Plants or Vegetables. | Musts or Worts. | Flags or | Algæ | Acolyledones vel Cellulosæ. | Plantæ vel Vegetabilia. |
| | | Mushrooms or | Fungi | | |
| | | Mosses or | Musci | | |
| | Leas or Herbs. | Palms, Lilies, &c. or | Palmites[a] | Monocotyledones vel Endogenæ. | |
| | | Grasses and Sedges or | Segetes | | |
| | | Ferns or | Filices | | |
| | Cresses or Plants. | Pines and Zamias or | Za-Pini[b] | Dicotyledones vel Exogenæ. | |
| | | Cresses, Frules, or | Eucarpæ[c] | | |
| | | Sel-worts | Selanthi[d] | | |

It is curious to observe the coincidence which exists between the popular and scientific distribution and nomenclature, and that the Fungi, Algæ, Musci, Filices, Segetes, &c. are but technical synonymes for nearly equivalent groups, known to all as mushrooms, flags, mosses, ferns, grasses, sedges, and so forth: and this is still further remarkably the case in the

[a] Including the *Petaloid Endorhizæ* of Richard, or Monocotyledons of Jussieu.

[b] Including the *Synorhizæ* of Richard.

[c] Including the *Exorhizæ* of Richard.

[d] Including the *Cytineæ* and *Rhizantheæ* of authors.

*Primary Groups admitted by Linnæus.*

| | | | | |
|---|---|---|---|---|
| Vegetabilia. | Monocotyledones vel Fruges. | Palmæ or | Palms, | (Principes.) |
| | | Gramina or | Grasses, | (Plebeii.) |
| | | Lilia or | Lilies, | (Patritii.) |
| | Dicotyledones vel Plantæ. | Herbæ or | Herbs, | (Nobiles.) |
| | | Arbores or | Trees, | (Proceres.) |
| | Acotyledones vel Cryptogama. | Filices or | Ferns, | (Novaccolæ.) |
| | | Musci or | Mosses, | (Servi.) |
| | | Algæ or | Flags, | (Vernaculi.) |
| | | Fungi or | Mushrooms, | (Nomades.) |

I stop not here to justify the classes and the regions into which the vegetable reign, or kingdom, has been almost universally distributed: this will engage our subsequent attention. Neither am I called on to defend the names which custom has, in general too loosely, applied thereto, and which science often, too fastidiously, condemns. Much of the arrangement I think very natural, and most of the terms, if used with somewhat more precision, highly expressive; (and why should not English technicalities be as carefully defined as Greek and Latin words?) and, although not all so classically elegant as some of our botanical nomenclature is, they are equally intelligible, and far more euphonious than many semi-barbarous technicalities: but of this, more hereafter. I merely use the vulgate now, because, although I am aware that many of my hearers are well versed in botanical language, to some it may not be familiar; and hence, that every point may be explained, it is, as I have already observed, convenient to assume, even if it be a kind of legal fiction, that all are ignorant of the subject. Hence, those who are veterans in botany will be pleased to excuse, (indeed, I know they will ever be the last to condemn,) the adoption of any method which may tend to familiarise science and facilitate the progress of the student. Who is there that has not at some time felt the galling yoke of technicalities? Who has not found, that to learn a science, and at the same time to learn a language, is indeed to have the tale of bricks demanded, while the straw to make them is denied.

Let me, therefore, in conjunction with the scientific names, employ the common English synonymes, whenever such exist, and, when there are none known, translate the foreign epithets into our mother tongue; at least for the purposes of this momentary glance at the several most commonly accepted sections of the vegetable world, which, by means of specimens and drawings, can be brought as it were in review before us.

The first class to which I shall direct your attention contains several very curious types of plants which, collectively, are termed *Flags,* or *Algæ.* Their English name has reference to the flagging habits of a large proportion; as, for example, the sea weeds, which are usually fixed to rocks and stones, and flag, i. e. droop or float, according as the water quits them or bears them up. *Alga,* the technical synonyme, is said to be derived from *ab algore,* coldness, as if it had been supposed that some of these productions, which are chiefly aquatic, were formed by the congelation of films or drops of water; to which, indeed, many bear no slight resemblance. To this ancient hypothesis there seems to be allusion in the names of several: e. g. *ulva* (ab uligine,) signifies ooziness or moisture: as our English word *laver,* from *laver* (a lavo) to wash, literally means *froth, scum,* or *lather*; whence, perhaps, lāver or lăver. Again, Halymenia, if literally translated, gives a pellicle of sea-water or sea-membrane. Thus, also, *Achnanthes* is sea-froth; *Anthachne,* froth-flower; and *Alcyonidium,* the foam of the sea. Many other similar examples might be given, for they continually occur, and in every language; although, when veiled in foreign tongues, or even when custom has given them an adventitious meaning, their original significations are often not attended to. Thus our own *mildew* is but a contraction of *soft* or *mild dew,* referring to the delicate texture of the minute plants of which mildew consists, and of which each spot is, as it were, a forest.

In the infancy of philosophy, such fanciful speculations and ideas, which we now think absurd, were common to all branches of science, and to other departments of natural history, as well as to the study of plants. Indeed, it is comparatively not long since, that an elaborate and learned dissertation was written in order seriously to prove that the "flowing gossamer," the aerial spider's web, so common in autumnal mornings, is *not* frozen dew.

The names alone are all that now happily remain to us of many of these crude doctrines which we are too apt to denominate absurdities, not remembering that many of our received hypotheses, it is more than probable, are equally destitute of truth. These are but the clouds which attend the morning twilight of philosophy; and, as the sun of science rises, like the early dew they pass away.

The class, denominated the Flags or Algæ, includes in its several orders, sections, types, and genera, some of the most curious living structures which as yet are known: protophytes just emerging from lifelessness to life, and beings which are almost animals, but still linger on the confines of the vegetable world.

Many of these microscopic creatures are so simple in their nature, that their very simplicity renders them a doubt. Here, indeed, is the problem of which mention has been already made; for so similar are many of the lower tribes of algæ and of fungi, that it is not only sometimes indeterminable to which of these two great classes certain individuals should be referred, but whether, in truth, they are plants at all; for, strange as the statement does appear, many of them may only be parts of other organic beings, and to more there has been attributed an animal existence.

Upon this point, however, modern research has thrown very considerable and very important light; and several of those ambiguous things called infusorial animalculæ, and named and arranged as such in their systems by zoologists, and to which, by some, an equivocal or fortuitous generation had been most gratuitously attributed, it is more than probable are not of an animal, but of a vegetable, nature: and, besides this, very many of the moving corpuscules, which have often been mistaken for monads, and which hence were once most unphilosophically supposed to have sprung into existence without parental aid, are proved to be merely portions of dissolved or dissolving organic matter, loosened in its structure, and put into motion by physical powers, which had previously escaped detection by the observant eye of man. I allude, of course, to the curious phenomena described by Porrett under the name of *Electro-filtration*, and which Dutrochet has termed *Endosmose* and *Exosmose*, i. e. a *flux inwards* and a *flux outwards*, from the circumstance of two currents of different strengths being noticed to pass through organic membranes, when the fluids on either sides are of different densities or in different electrical states; and which will either fill or empty a fixed saccule, or put a moveable one in motion. This phenomenon was first observed by Dutrochet to take place in the cellules of a small conferva, or moss-like production, which he detached from a fish's tail. Each portion of this moss consisted of a filament and saccule, from which globules were expelled, and into and out of which the currents of fluids passed. He procured other similar globules by putting pieces of flesh into the water, so that their formation was not connected with the living state of the fish. He saw these globules spread throughout the fluid, agitate themselves in divers directions for an instant, and then precipitate themselves to the bottom of the vessel.

But methinks I see some ultra-utilitarian smiling at the thought of a grave philosopher being thus engaged for hours in watching the motions of a corpuscule so minute as to be scarcely visible to the naked eye, and methinks I hear him ask "cui bono?" a question that any child may ask, but one that the wisest philosophers must often find it difficult to answer; although they may be far from admitting the pertinency of the question. When such queries are proposed, as they often are, I love to meet them with Franklin's counter question, "What's the use of a baby?" for no one will venture to inquire, what is the use of a man?

The experiments which have led to this digression as yet are in their infancy; but, even imperfect and crude as they confessedly at present are, they have already thrown much light on some very obscure parts of animal and vegetable physiology, and they promise to afford much more: they certainly disclose one of the most curious physical forces which have been discovered in modern times, and the just value of which we have not at present the means of estimating.

The same observations apply, and perhaps with still more truth, to that most curious discovery lately made by the celebrated Mr. Robert Brown, who has shewn, by an unexceptionable series of experiments, that locomotion, even when apparently independent of external forces, may and does exist among particles that are absolutely lifeless; nay, which have never been alive: so that, should not this phenomenon admit some more probable solution, it would seem that the long-established definition, which declares matter to be inert, may perhaps require a serious modification.

This apparently independent motion of the molecules of matter may appear to some to be a close approximation to the vital motions of plants, or the spontaneous movements of animals; and, indeed, the idea would seem more plausible than the belief of some German philosophers, that crystallization is an effect of vitality. The facts are simply these: that grains of pollen, particles of dead plants, some of which have been in herbaria for upwards of a century, nay, even fragments of pounded glass and stone, when diffused through water, and viewed with a good microscope, are seen to be in a constant state of motion; and this independent of any evaporation of, or currents in, the fluid. Furthermore, that they still maintain their restless activity when hermetically sealed between two plates of glass, so as to exclude, as far as possible, all external agitation; and are found, even under such circumstances, to continue their motions unremittingly during an indefinite period; nay, even after the lapse of months, (I believe we may now say years,) to be as full of motion as when first observed.

This discovery, as just now hinted, has been thought by some to militate against the ancient dogma, which enunciates the inertia of matter. It would ill become me to advance any speculations other than as mere hypotheses, and this the more especially as the discoverer himself, with that modesty which always attends true genius, does not even venture an explanation: I, therefore, scarcely dare to suggest that it would be desirable to ascertain whether these movements may not be indicators of external motions, so slight as to be imperceptible to other means, rather than as inherent in the particles themselves. Just as many atmospheric changes are notorious with the water, that are utterly inappreciable with the mercurial barometer; and as the expansions of bodies by heat, and the vibrations of sound, are measurable by some instruments, which are imperceptible by others, so it would be desirable to ascertain whether the motions of these molecules do or do not depend upon vibrations, otherwise imperceptible, communicated by distant moving bodies on the surface of the earth, to the supports on which they stand; or whether it is possible, as the movements seem very constant and similar, that they can evidence the motions of the earth itself, and thus afford the means of constructing a delicate *kineometer*.

But, although many pseudo-*animalculæ* and (if we may be allowed the parallel word) *vegetalculæ* (?) are thus shewn not to be those wonders they were once supposed to be, and although locomotion is thus proved not to be absolutely diagnostic of life, still they are not the less wonderful now that they are regarded as what they truly are, lifeless corpuscules, put in motion by newly discovered and extraordinary laws, which their observation has been the first to reveal, than when they were considered paradoxes, and almost a reproach to natural science. And besides, even after their exclusion from the organic realm, there still remain many living beings, as simple in their structure and as curious in their functions as imagination can well conceive a vital organization to be, or ever to have been. For example, the slimy matter often seen on rocks and stones, on hard gravel walks, and on damp walls and cellars, or on the glass of windows, garden pots, and so forth, and which is often so minute as to be lost to ordinary vision, consists of curious and most admirable vegetable structures. All the green pulverulent coating seen on old trees and paling is likewise found, by microscopic observations, to be composed of an infinite number of small plants, of an exceedingly primitive formation.

The slimy masses known as Will o' the wisps, or Nostocs, are instances of other similar species, some of which are called by the common people "flowers of heaven;" a name which they deserve more than many that are often given to plants, if it be true, as the old herbalists declare, that, "infused in brandy, they cause a disgust to that liquor in those who drink of it;" for, as Johnston adds, they would then become "an excellent remedy for the '*potatores summi.*'"

Not one of the least curious of the lowly flags is the "red snow," which excited so much attention on Captain Ross's return from the North Pole in 1819. This phenomenon seems in some cases to depend upon the sudden appearance of a very minute plant, which the microscope declares to consist of small cells filled with a red fluid, and which is referred to a genus named, from its very simple structure, "*proto-coccus.*"

This plant, as well as the Palmella cruenta, or gory dew, Lepraria kermesina, or bloody rain, with many others called *reeks* or *earth-sweats*, as well as certain minute animalculæ, suddenly appear, and in such great abundance as even to tinge pools of water with the hue of blood, to make red stains on the sea shore, and to discolour considerable tracts of ground, so as to simulate red snow, or dew, or rain; and such, in fact, the appearance is vulgarly supposed to be. These occurrences are often regarded by the ignorant as of sinister omen: indeed, whole towns have been occasionally alarmed with the report that, in the course of a single night, the water of their pools had become changed to blood; and the dismay was not relieved until a philosopher exhibited to the eyes of many, the minute living corpuscules which had wrought the change of hue, and which were easily separable by filtering the fluid.

Palmella cruenta, or gory dew, is common in many places: I found it abundantly a few weeks since at Oxford, and it is frequently observed in damp situations, forming "broad indeterminate patches of a deep rich purple, with a shining surface, as if blood or red wine had been poured over the stone or ground." During dry weather, it contracts, grows dull, and disappears; but, after rain, spreads anew, resumes its sanguine colour, and becomes conspicuous even to vulgar gaze. Its history affords (says Johnston, in his Berwick Flora,) an easy explanation of a phenomenon considered supernatural by monkish chroniclers, and to which Drayton, in his notes to Polybion, refers: "In the plain near Hastings, where the Norman William, after his victory, found King Harold slain, he built Battle Abbey, which at last (as divers other monasteries) grew to a town enough populous. Thereabout is a place which, after rain, always looks red, which some have attributed to a very bloody sweat of the earth, as crying to heaven for revenge of so great a slaughter."

